# Spontaneous Regression of a Pulmonary Inflammatory Myofibroblastic Tumor

**DOI:** 10.7759/cureus.78767

**Published:** 2025-02-09

**Authors:** Branden Wilson, John Allonce, Kshitij Mehrotra, Dali Huang, David H Lindner

**Affiliations:** 1 Pulmonology Disease and Critical Care, NCH (Naples Comprehensive Health) Healthcare System, Naples, USA; 2 Internal Medicine, NCH (Naples Comprehensive Health) Healthcare System, Naples, USA; 3 Pathology, NCH (Naples Comprehensive Health) Healthcare System, Naples, USA

**Keywords:** aspetic inflammatory mass, inflammatory myofibroblastic tumor, lung biopsy, malignancy, mesenchymal tumor, pulmonary inflammatory myofibroblastic tumor, rare lung disease, spindle cell lesion, spontaneous resolution, thoracic

## Abstract

Inflammatory myofibroblastic tumors are rare benign mesenchymal neoplasms composed of myofibroblastic cells intermixed with an inflammatory infiltrate of cells including plasma cells, lymphocytes, and histiocytes. These tumors are commonly seen in children and are rare in adults. Management of these tumors is challenging due to their unpredictable behavior. Here, we present a rare case of a pulmonary inflammatory myofibroblastic tumor that regressed spontaneously following a minimally invasive computed tomography-guided percutaneous lung biopsy.

## Introduction

Inflammatory myofibroblastic tumor (IMT) is a rare neoplasm consisting of myofibroblastic and fibroblastic spindle cells accompanied by an inflammatory infiltrate [1]. It was first reported in the lung in 1939 [1]. IMTs occur with equal frequency in both male and female individuals and can develop in any organ [1-3]. Pulmonary IMTs are more commonly found in children and adolescents but can also occur in adults [1,2]. Pulmonary IMTs currently make up 20% of primary pulmonary tumors in children and less than 1% of adult primary pulmonary tumors [1-3].

The clinical presentation of pulmonary IMTs can vary widely; some patients may be asymptomatic, while others may present with cough, shortness of breath, chest pain, constitutional symptoms, or weight loss [1-4]. Pulmonary IMTs have been associated with other diseases, including autoimmune disorders like IgG4 syndrome and Sjogren's disease [1-3]. They have also been reported in association with infections, including pulmonary *Mycobacterium tuberculosis*,* Moraxella, *and *Pseudomonas* pneumonia, as well as viral infections with Epstein-Barr virus (EBV) andhuman herpesvirus 8 (HHV-8) [1]. These associations suggest that immune dysregulation, whether caused by infections or autoimmune disorders, may play a role in the development of IMTs. Radiographically, pulmonary IMTs often appear as a well-defined, round, or oval mass, typically found in the lower lobes, peripheral lung parenchyma, and subpleural areas [3]. Diagnosis is difficult without tissue biopsy [1-4].

Management is complicated due to its unpredictable course; while most lesions remain benign, others can grow in size, invade nearby tissues, and metastasize [1-7]. Surgical resection is generally recommended due to the potential for growth and spread [1-6]. Medical therapies such as chemotherapy, radiation, and corticosteroid therapy have also been reported in the literature [1,4-8]. Here, we present a rare case of spontaneous regression of a pulmonary IMT following a minimally invasive computed tomography (CT) guided percutaneous lung biopsy, along with a brief review of this rare phenomenon seen in pulmonary IMTs.

## Case presentation

**Figure 1:**
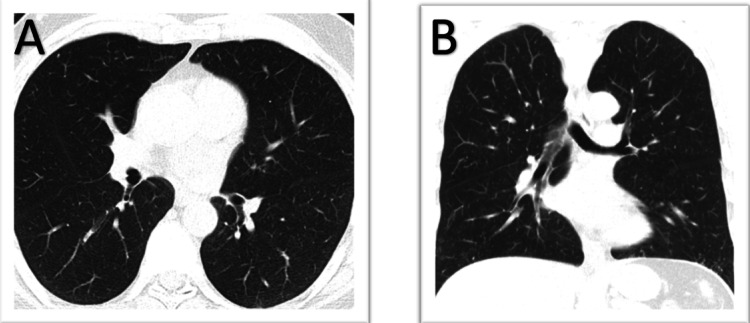
Baseline chest CT (A) axial and (B) coronal views showing subcentimeter pulmonary nodules in the anterior right and left upper lobes with reticulonodular changes in bilateral lower lobes

A 67-year-old male patient with a past medical history of hyperlipidemia and a family history of coronary artery disease was referred to the pulmonary clinic for evaluation of abnormal CT of the chest. Five months prior, he underwent a calcium-scoring CT of the chest, which incidentally revealed fibrotic and reticular changes in bilateral lower lobes, along with small subcentimeter pulmonary nodules in both upper lobes (Figure 1). He denied any cough, shortness of breath, chest pain, unintentional weight loss, or constitutional symptoms. Additionally, he reported no joint pain, rashes, dry eyes, or history of autoimmune disease. He did not report any upper respiratory or other infectious symptoms in the months prior to his presentation. He described a smoking history spanning two years when he was younger. He denied any occupational exposures including asbestos, silica, mining, welding, or radon exposure. There was no family history of lung disease or lung cancer. 

**Figure 2:**
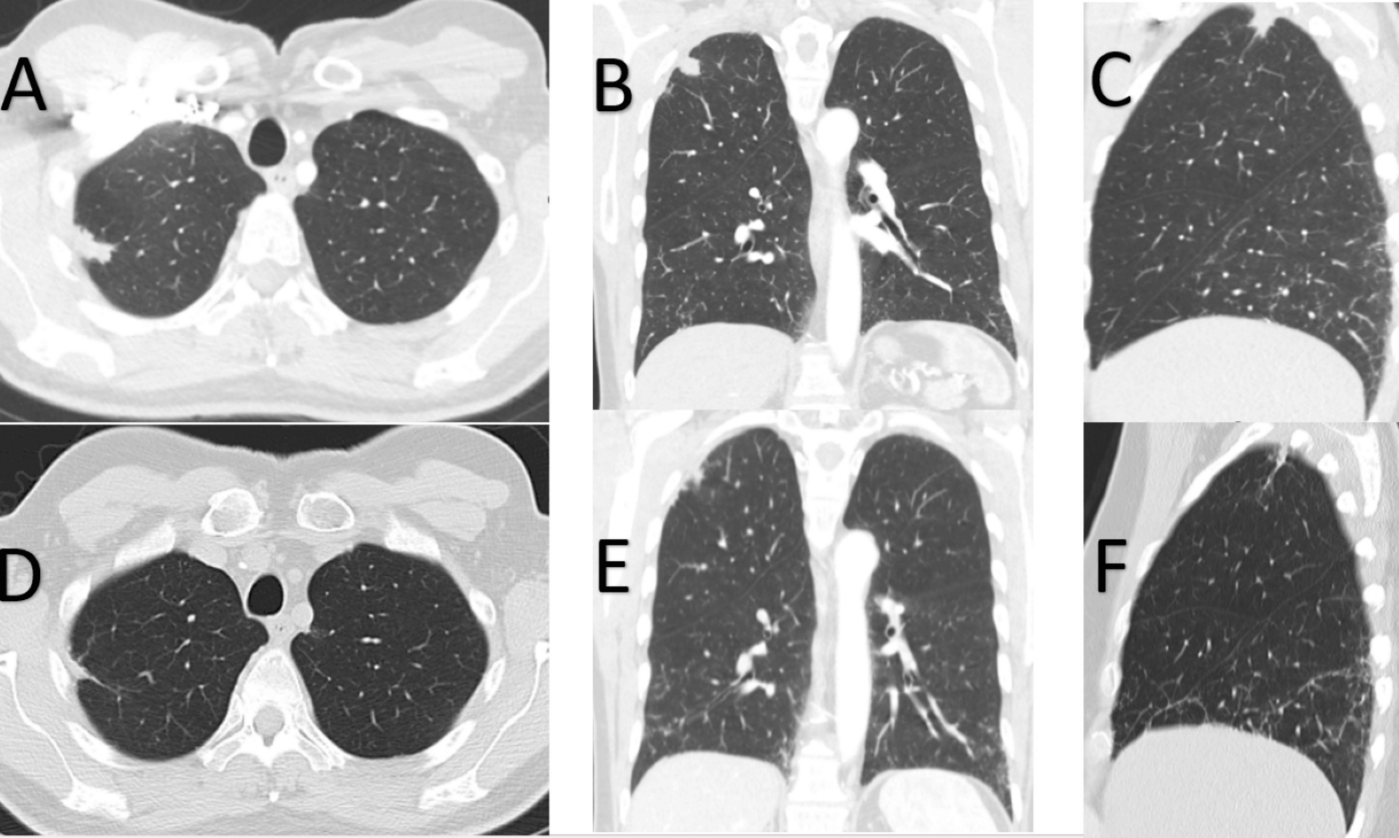
Initial six-month follow-up chest CT (A) axial, (B) coronal, and (C) sagittal views showing a new mass measuring 3.2 cm x 2.1 cm at the lateral aspect of the right pulmonary apex, contiguous with adjacent thickened pleura with reticulonodular and fibrotic changes in the bilateral lower lobes; Six-month follow-up post-lung biopsy chest CT (D) axial, (E) coronal, (F) sagittal views revealing regression of the lesion, with only chronic scarring observed from the prior lung biopsy, and stable reticulonodular and fibrotic changes in the bilateral lower lobes.

A follow-up dedicated CT of the chest, performed six months after the initial scan, revealed a new mass measuring 3.2 cm x 2.1 cm at the lateral aspect of the right pulmonary apex, contiguous with adjacent thickened pleura with reticulonodular and fibrotic changes in bilateral lower lobes (Figures 2A-2C).

**Figure 3:**
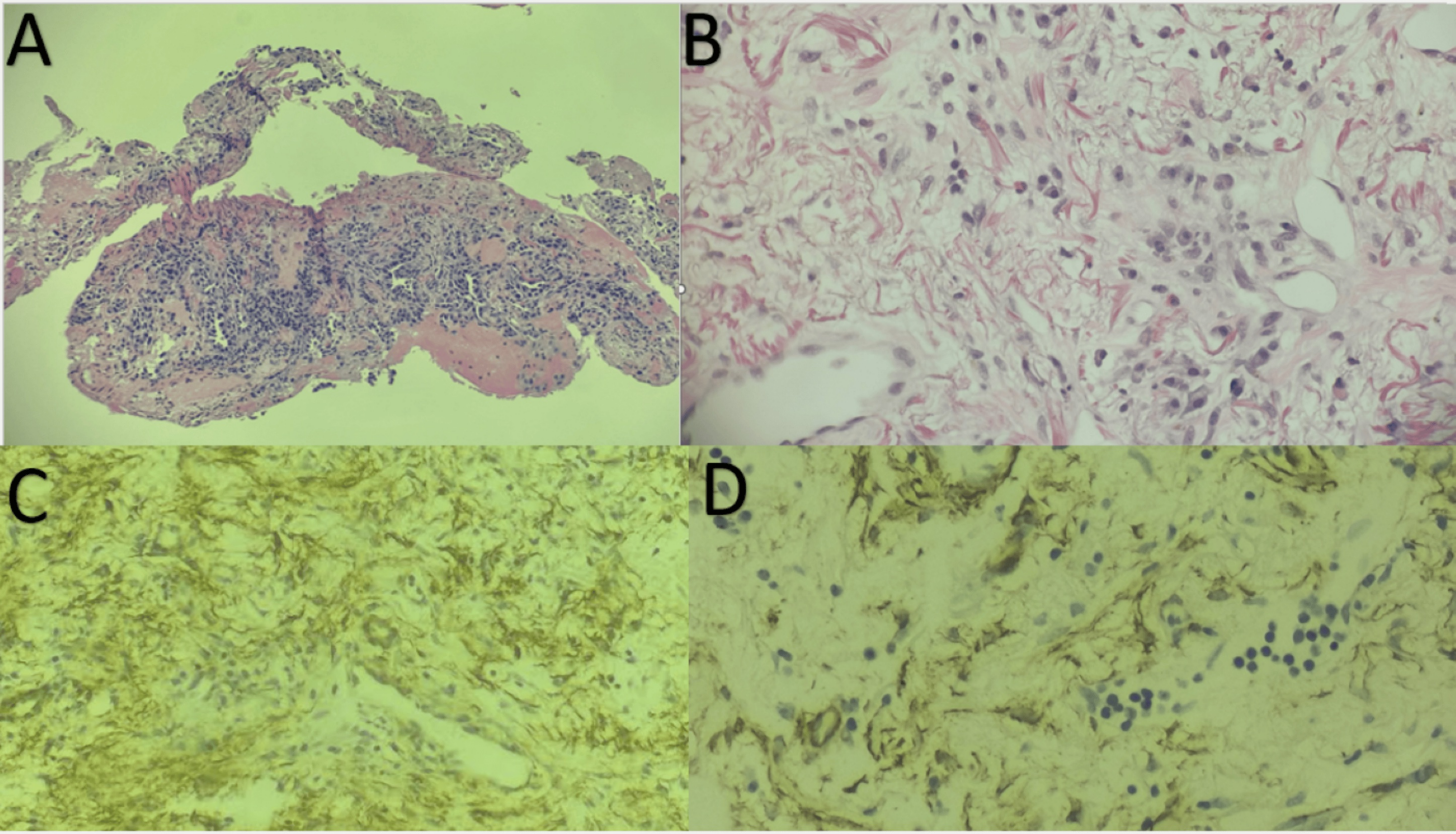
Photomicrographs of CT-guided percutaneous lung biopsy specimen (A) and (B): Hematoxylin and eosin staining showing a benign spindle cell mesenchymal process with spindle myofibroblastic cells in a background of plasma cells, eosinophils, and occasional lymphocytic infiltrates consistent with an inflammatory myofibroblastic tumor. (C) and (D): Immunostaining for vimentin and smooth muscle actin were diffusely positive and were negative for anti-cytokeratin AE1/AE3 and CD34.

The patient underwent minimal invasive CT-guided percutaneous lung biopsy, which revealed a benign spindle cell mesenchymal process with scattered lymphocytes, plasma cells, and eosinophils consistent with an IMT. Immunostaining was diffusely positive for vimentin and smooth muscle actin and was negative for anti-cytokeratin AE1/AE3, and CD34 (Figure 3). No pathogens were detected on tissue culture. Anaplastic lymphoma kinase (ALK) staining was not performed. 

After extensive discussion with the patient regarding the management of his pulmonary IMT, he decided on regular CT scan monitoring instead of surgical resection. He expressed his understanding of the risk of recurrence and metastasis without surgical intervention. Follow-up visits were scheduled every six months in which symptoms and repeat imaging were discussed and completed. Follow-up CT of the chest six months after lung biopsy revealed regression of the lesion, with only chronic scarring observed from the prior lung biopsy (Figures 2D-2E). He continued with yearly follow-ups with repeat CT chest imaging showing no new recurrence for the subsequent four years. During all his follow-up visits, he denied any cough, shortness of breath, chest pain, or unintentional weight loss.

## Discussion

Pulmonary IMTs histologically are characterized by a spectrum of myofibroblastic spindle cell proliferation, intermixed with an inflammatory infiltrate primarily composed of plasma cells, histiocytes, eosinophils, and lymphocytes [4,6-12]. Three distinct histologic patterns are recognized in the literature: (i) Plasma cell variant, which is characterized by inflammatory myxoid proliferation, exhibiting fascicles of spindled fibroblasts or myofibroblasts, minimal collagenous tissue, and abundant lymphocytes and plasma cells, (ii) Fibrohistiocytic type, which exhibits a spindle-cell pattern resembling a fibrous histiocytoma, with a proliferation of fibroblasts and myofibroblasts along with polyclonal plasma cells, xanthoma cells, and occasional giant cells, and (iii) Organizing pneumonia type, which exhibits a pattern with low cellularity, characterized by dense collagen and few spindle cells [4,6-12]. This histological diversity has led to its classification under various terms, including inflammatory pseudotumor, fibrous xanthoma, plasma cell granuloma, pseudosarcoma, and inflammatory fibrohistiocytic proliferation [4,6-12]. The various names used for this inflammatory neoplastic disorder highlight our limited understanding of its pathogenesis. Histopathological examination in the present case revealed a matrix of benign spindle cells intermixed with an inflammatory infiltrate of scattered lymphocytes, eosinophils, and plasma cells, consistent with the plasma cell variant.

The classification of IMT as a neoplasm has been debated over time [7,10-12]. This is likely due to the fact that proliferating myofibroblastic cells often exhibit no cellular atypia, necrosis is rare, and mitotic figures are infrequent. Recently, clonal gene rearrangements have been reported leading some to reconsider IMTs as a true malignant neoplasm [7,10-12]. The most frequently reported rearrangement affects the oncogenic ALK receptor tyrosine-kinase gene locus [7,10-11]. Translocations involving ALK result in a tyrosine kinase that remains constitutively active, as observed in other neoplasms such as lymphomas and non-small cell lung cancer. Up to 40-70% of all IMTs have been reported to have ALK overexpression [7,10-11]. ALK-negative IMTs have a higher reported frequency of metastasis compared to ALK-positive tumors. Other translocations have been reported in the *ROS1* gene, a receptor tyrosine kinase of the insulin receptor family [12]. While IMTs are usually slow-growing and maintain a stable size, they can potentially invade adjacent structures like the mediastinum, diaphragm, heart, and chest wall [7-10]. Moreover, IMTs exhibit a recurrence rate as high as 25%, reinforcing their classification as true malignancies [7-10].

Managing pulmonary IMTs is challenging due to its unpredictable progression and lack of established guidelines for diagnosis and treatment. Complete resection offers a favorable prognosis and reduces the risk of recurrence compared to no surgery [3-9]. It is a common practice to proceed with conservative resection to provide overall better outcomes [5-10]. While surgical resection offers high curative rates, there remains a question of whether alternative treatments or surveillance should be considered. Our patient chose to pursue surveillance instead of resection or other treatments. Remarkably, his right upper lobe mass spontaneously regressed following the CT-guided lung biopsy, with no recurrence observed to date.

Spontaneous regression of pulmonary IMTs has been documented in only seven other cases [13-18]. Radiologically, all seven cases of spontaneous regression presented with solitary masses, one with eccentric calcifications and another involving central cavitation. Additionally, two cases showed bilateral nodules [15,17]. Our patient presented with a peripherally right upper lobe solitary mass with associated pleural thickening.

Most patients with pulmonary IMTs are symptomatic at the time of presentation, with symptoms often related to the size or location of the tumor [13-17]. Interestingly, all the cases of spontaneous regression of pulmonary IMTs experienced regression of the tumor following an invasive procedure [13-18]. In 1981, Mandebeaum et al. documented two cases of spontaneous regression of a pulmonary IMT [13]. Their first case involved a 57-year-old man with a significant smoking history who presented with cough and dyspnea. Imaging showed a right hilar mass with eccentric calcification. He underwent thoracotomy with lung biopsy, which revealed benign plasma cells, fibrous tissue, and alveolar histiocytes typical of a pulmonary IMT. The patient was closely monitored over the next ten years, during which the mass resolved spontaneously. Their second case was that of a 38-year-old woman with chest pain and cough, diagnosed with a noncalcified right upper lobe density extending into the mediastinum. Following a diagnostic open anterior thoracotomy with biopsy, revealing a large yellowish-white tumor (3 cm x 4 cm), the patient experienced complete regression of both pulmonary and mediastinal lesions over a five-year period [13].

Maurya et al. reported the case of a 44-year-old male smoker presenting with chronic cough, hemoptysis, fever, and weight loss [14]. CT of the chest showed a solitary homogenous soft-tissue mass with cavitation and surrounding consolidation in the right middle lobe. An inconclusive CT-guided biopsy was followed by a wedge biopsy, confirming inflammatory cells predominantly lymphoplasmacytic cells consistent with a pulmonary IMT. Six months later, subsequent imaging showed only a residual scar at the biopsy site.

Only two other cases have reported spontaneous regression following a minimally invasive procedure such as CT-guided lung or transbronchial biopsy, similar to our patient [15,16]. Checrallah et al. described a 65-year-old man with cough, shortness of breath, and hemoptysis, found to have bilateral ill-defined nodular densities on CT imaging [15]. A CT-guided core lung biopsy confirmed an inflammatory pseudotumor, with complete regression of the pulmonary lesions within three months and sustained regression over seven years. Sakashita et al. reported a 72-year-old man presenting with cough, fever, and weight loss who was found to have a round tumor in the left lower lobe on a chest X-ray [16]. Transbronchial lung biopsy demonstrated histiocytic and lymphocytic infiltration in the alveolar space with spindle myofibroblasts and plasmacytes, consistent with an inflammatory pseudotumor. Four weeks post-biopsy, a follow-up x-ray showed regression of the pseudotumor.

Only one case of spontaneous regression in a child has been reported [17]. A previously healthy nine-year-old presented with unintentional weight loss and was found to have a 4.5 cm mass in the right lung. After undergoing a lung biopsy, the patient was managed conservatively with close monitoring. Follow-up CT chest imaging six weeks later showed significant regression of the lesion, and by seven months, the lesion had completely resolved.

Our patient was asymptomatic at presentation and remained so during pulmonary follow-up, a rarity as most cases present with infection-like symptoms. This has been reported only once earlier by Tanabe et al., where a 62-year-old male patient presented asymptomatically with multiple bilateral pulmonary nodules on CT imaging [18]. The patient underwent bronchoalveolar lavage and transbronchial lung biopsies which were negative for infection and non-diagnostic. Subsequently, the patient underwent a video-assisted thoracic surgical (VATS) lung biopsy of one of the nodules, revealing marked inflammatory cell infiltration and fibrosis. Plasma cells were predominant components of the infiltrating inflammatory cells and were found to be IgG4 positive. On the one-month follow-up following VATS multiple nodules spontaneously disappeared without any treatment or medication [18]. 

IgG4 plasma cell proliferation is crucial in the regulatory and anti-inflammatory responses that support tissue repair and regeneration [19]. IgG4 plasma cell proliferation is reported in two cases of spontaneous regression seen in pulmonary IMTs [16,18]. A potential immune trigger may lead to the recruitment and proliferation of IgG4 plasma cells, which could be associated with the spontaneous regression observed in pulmonary IMTs [16,18,19]. This immune response could be triggered by infection, trauma from lung biopsy, or an autoimmune process such as IgG4-related disease. Interestingly, no documented infections were reported in our patient or in any of the seven cases of spontaneous regression [13-18]. This immune response may represent an inflammatory reaction induced by the trauma of the lung biopsy, recruiting other immune cells and facilitating tissue remodeling, ultimately leading to the spontaneous regression seen in pulmonary IMTs.

In a systematic review involving 38 patients with intra-abdominal IMTs who underwent biopsy and were managed conservatively without surgery, spontaneous regression was observed in 17 cases [20]. The traumatic nature of abdominal biopsies performed in these patients may similarly trigger an innate inflammatory response, leading to the spontaneous regression of the IMT, similar to observations in pulmonary cases. Spontaneous regression was more frequently noted among middle-aged and older patients. Our patient falls into the older age group, consistent with the majority of other cases of complete regression of pulmonary IMTs reported in the literature. Therefore, age may serve as a positive prognostic marker, as observed in intra-abdominal cases [20].

Histologically, among the seven cases of pulmonary IMT along with the present case, the plasma cell variant was the most common and was described as a benign spindle myofibroblastic process interspersed with an infiltration of inflammatory cells [13-18]. This suggests that the plasma cell variant may have a better prognosis and potential for spontaneous regression, warranting further investigation. Unfortunately, ALK rearrangements were not reported in any of the cases of spontaneous regression, including ours. ALK-positivity in pulmonary IMTs could represent another plausible reason for spontaneous regression, given their better prognostic value observed across all types of IMTs [9-11]. Future cases should investigate ALK translocation and other gene rearrangements to gain further insight into the rarity of spontaneous regression in pulmonary IMTs. Long-term follow-up of reported cases of spontaneous regression in pulmonary IMTs is crucial to enhance understanding of this subtype's natural history, especially considering reported recurrences years after resection.

## Conclusions

IMTs of the lung present a unique clinical challenge due to their varied presentation and progression. Despite the common approach of surgical resection, our patient's decision for surveillance over a four-year period resulted in the spontaneous regression of the tumor without any recurrence to date. This outcome highlights an alternative in the management of certain pulmonary IMTs, suggesting that with careful monitoring, conservative management may be a viable option for some patients. Our case adds to the growing body of evidence that alternative management strategies, including vigilant observation, may be effective and less invasive.

Further studies and case reports are needed to better understand the natural history of pulmonary IMTs and to identify which patients might benefit from non-surgical approaches. This case contributes to the limited literature on the successful non-operative management of pulmonary IMTs and encourages a re-evaluation of treatment protocols, potentially incorporating more conservative approaches where appropriate.

## References

[1] Khatri A, Agrawal A, Sikachi RR, Mehta D, Sahni S, Meena N (2018). Inflammatory myofibroblastic tumor of the lung. Adv Respir Med.

[2] Lai LM, McCarville MB, Kirby P (2015). Shedding light on inflammatory pseudotumor in children: spotlight on inflammatory myofibroblastic tumor. Pediatr Radiol.

[3] Ufuk F, Herek D, Karabulut N (2015). Inflammatory myofibroblastic tumor of the lung: unusual imaging findings of three cases. Pol J Radiol.

[4] Eyden B, Banerjee SS, Shenjere P, Fisher C (2009). The myofibroblast and its tumours. J Clin Pathol.

[5] Cerfolio RJ, Allen MS, Nascimento AG, Deschamps C, Trastek VF, Miller DL, Pairolero PC (1999). Inflammatory pseudotumors of the lung. Ann Thorac Surg.

[6] (2004). Pathology and Genetics of Tumours of the Lung, Pleura, Thymus and Heart. Pathology and Genetics of Tumours of the Lung, Pleura, Thymus and Heart.

[7] Lawrence B, Perez-Atayde A, Hibbard MK (2000). TPM3-ALK and TPM4-ALK oncogenes in inflammatory myofibroblastic tumors. Am J Pathol.

[8] Watanabe R, Ano S, Kikuchi N, Saegusa M, Shigemasa R, Kondo Y, Hizawa N (2024). Inflammatory myofibroblastic tumor directly invading the right first rib treated with oral steroids: a case report. BMC Pulm Med.

[9] Gal AA, Koss MN, McCarthy WF, Hochholzer L (1994). Prognostic factors in pulmonary fibrohistiocytic lesions. Cancer.

[10] Chan JK, Cheuk W, Shimizu M (2001). Anaplastic lymphoma kinase expression in inflammatory pseudotumors. Am J Surg Pathol.

[11] Pearson AD, Barry E, Mossé YP (2021). Second Paediatric Strategy Forum for anaplastic lymphoma kinase (ALK) inhibition in paediatric malignancies: ACCELERATE in collaboration with the European Medicines Agency with the participation of the Food and Drug Administration. Eur J Cancer.

[12] Mai S, Xiong G, Diao D, Wang W, Zhou Y, Cai R (2019). Case report: crizotinib is effective in a patient with ROS1-rearranged pulmonary inflammatory myofibroblastic tumor. Lung Cancer.

[13] Mandelbaum I, Brashear RE, Hull MT (1981). Surgical treatment and course of pulmonary pseudotumor (plasma cell granuloma). J Thorac Cardiovasc Surg.

[14] Maurya V, Aditya Gupta U, Dewan RK, Jain S, Shah A (2013). Spontaneous resolution of an inflammatory pseudotumour of the lung subsequent to wedge biopsy. Arch Bronconeumol.

[15] Checrallah A, Riachi M, Slaba S (2005). Inflammatory pseudotumors of the lung with spontaneous regression [Article in French]. J Med Liban.

[16] Sakashita K, Takamori M, Murata K, Wada A, Fujita A, Enatsu K (2011). A case of IgG4-positive inflammatory pseudotumor which rapidly resolved [Article in Japanese]. Nihon Kokyuki Gakkai Zasshi.

[17] Brown KC, McCarthy VP, Gaines T (1998). Spontaneous resolution of a plasma cell granuloma in a 9-year-old. J Assoc Acad Minor Phys.

[18] Tanabe N, Kato M, Yonemoto C, Koshimo Y, Goto S, Kawashima M (2008). A case of IgG4-related inflammatory pseudotumor of the lung [Article in Japanese]. Nihon Kokyuki Gakkai Zasshi.

[19] Liu C, Zhang P, Zhang W (2020). Immunological mechanism of IgG4-related disease. J Transl Autoimmun.

[20] Zhao JJ, Ling JQ, Fang Y (2014). Intra-abdominal inflammatory myofibroblastic tumor: spontaneous regression. World J Gastroenterol.

